# Quantity and clinical relevance of circulating endothelial progenitor cells in human ovarian cancer

**DOI:** 10.1186/1756-9966-29-27

**Published:** 2010-03-24

**Authors:** Yajuan Su, Lei Zheng, Qian Wang, Weiqi Li, Zhen Cai, Shilong Xiong, Jie Bao

**Affiliations:** 1 Department of Clinical Laboratory, Nanfang Hospital, Southern Medical University, Guangzhou, PR China

## Abstract

**Background:**

Circulating bone marrow-derived endothelial progenitor cells (EPCs) have been reported to participate in tumor angiogenesis and growth; however, the role of circulating EPCs in tumor progression is controversial. The role of circulating EPCs in ovarian cancer progression and angiogenesis has not yet been investigated.

**Methods:**

The number of circulating EPCs in the peripheral blood in 25 healthy volunteers and 42 patients with ovarian cancer was determined by flow cytometry. EPCs were defined by co-expression of CD34 and vascular endothelial growth factor receptor 2 (VEGFR2). In addition, we determined CD34 and VEGFR2 mRNA levels by real-time reverse transcription-polymerase chain reaction. Plasma levels of vascular endothelial growth factor (VEGF) and matrix metalloproteinase-9 (MMP-9) were determined by enzyme-linked immunosorbent assay.

**Results:**

Circulating levels of EPCs were significantly increased in ovarian cancer patients, correlating with tumor stage and residual tumor size. Higher levels of EPCs were detected in patients with stage III and IV ovarian cancer than in patients with stage I and II disease. After excision of the tumor, EPCs levels rapidly declined. Residual tumor size greater than 2 cm was associated with significantly higher levels of EPCs. In addition, high circulating EPCs correlated with poor overall survival. Pretreatment CD34 mRNA levels were not significantly increased in ovarian cancer patients compared with healthy controls; however, VEGFR2 expression was increased, and plasma levels of VEGF and MMP-9 were also elevated.

**Conclusions:**

Our results demonstrate the clinical relevance of circulating EPCs in ovarian cancer. EPCs may be a potential biomarker to monitor ovarian cancer progression and angiogenesis and treatment response.

## Introduction

Ovarian cancer is one of the most aggressive gynecological malignancies, and its high mortality is most often a direct result of delayed diagnosis. Only 25% of ovarian cancers are diagnosed while the malignancy is still confined to the ovary, and the cure rate in these patients can reach 90%. The remaining 75% of ovarian tumors have spread beyond the ovary by the time of diagnosis, and the cure rate for these patients is lower than 20% [1].

With the advent of molecular-targeted therapies, treatment for ovarian cancer is now moving beyond conventional chemotherapy. Inhibition of the specific cytokines essential for tumor vascularization is one such a therapy [2]; thus, anti-angiogenesis therapy has become a new strategy for ovarian cancer treatment. No proven biomarkers of tumor angiogenesis have been fully characterized; however, tumor microvascular density is used to predict tumor metastasis, recurrence, and prognosis. Determining microvascular density is a highly invasive procedure, and its association with the clinical outcome in ovarian cancer is uncertain [3,4]. For that reason, the development of noninvasive biomarkers would be useful to evaluate tumor angiogenesis and growth as well as the efficacy of antiangiogenic drugs in ovarian cancer.

Recent studies using various animal models of cancer have suggested a role for EPCs in tumor angiogenesis and growth [5,6]. EPCs are present in the peripheral blood; in response to certain signals or cytokines, their levels are elevated and they are recruited into the neovascular bed of the tumor [7]. Emerging evidence suggests that changes in EPC levels may predict the efficacy of anticancer drug combinations that include antiangiogenic agents [8]. Although these data suggest a relationship between EPCs and tumor angiogenesis, the exact role of these cells in the pathogenesis of ovarian cancer has not been completely elucidated.

The aim of this study was to determine the correlation between EPC levels and disease progression and angiogenesis in ovarian cancer. To that end, we quantified circulating EPCs from the peripheral blood of ovarian cancer patients by flow cytometry, before and after cancer treatment. In addition, we used real-time quantitative reverse transcription polymerase chain reaction (RT-PCR) to evaluate mRNA levels of EPC-specific markers CD34 and vascular endothelial growth factor receptor 2 (VEGFR2) in the peripheral blood of ovarian cancer patients. Plasma protein levels of vascular endothelial growth factor (VEGF) and matrix metallopeptidase-9 (MMP-9) were also determined.

## Materials and methods

### Patients

This study was approved by the local ethics committee, and informed consent was obtained from all study participants. Forty-two patients (median age, 43 years old; age range, 21-59 years old) with histologically proven ovarian cancer, including serous cancer (n = 23), mucinous cancer (n = 13), and endometrioid cancer (n = 6), were included along with a control group of healthy women (n = 25, age range, 18-35 years old). Tumors were classified according to the 1987 staging criteria recommended by the Federation of Obstetrics and Gynecology (FIGO). Of these patients, 30 patients underwent surgery for their malignancy, and 12 patients were treated with chemotherapy. These patients had no additional malignant, inflammatory, or ischemic disease, wounds, or ulcers that could influence the number of circulating EPCs. Peripheral blood samples of these patients were collected prior to treatment. All patients in this study received regular follow-up for 18 to 24 months (median follow-up, 20.2 months) after discharge. During this period, patients underwent physical examinations and related laboratory tests or imaging examinations once every 1 to 3 months. Blood samples were collected at 1 month after chemotherapy or surgery.

### Biological Samples and Flow Cytometric Analysis

Analysis was based on the expression of surface markers CD34 and VEGFR2 on cells in the mononuclear gate where EPCs are commonly found. CD34^+ ^and VEGFR2^+ ^are commonly used as markers for EPCs [9-11]. Peripheral blood was collected by venipuncture using ethylenediaminetetraacetic acid (EDTA) as an anticoagulant. Then, 100 μL whole blood was labeled with phycoerythrin (PE)-conjugated anti-human VEGFR2 and fluorescein isothiocyanate (FITC)-conjugated anti-human CD34 (BD, Franklin Lakes, NJ USA) by incubating for 30 min at 4°C according to the manufacturer's recommendations. Fluorescent isotype matched antibodies IgG1-FITC/IgG1-PE (BD) were used as controls. The suspension was then incubated with fluorescence-activated cell sorter (FACS) lysing solution (BD, Franklin Lakes, NJ USA) for 10 min, according to the manufacturer's instructions. After washing in phosphate buffered saline (PBS) and fixation in 1% formaldehyde, samples were analyzed on a FACSCalibur Instrument (BD). The percentage of double-positive mononuclear cells (CD34^+^/VEGFR2^+^) was converted to cells per ml of peripheral blood using the complete blood count (CBC).

### Quantitative real-time RT-PCR

To quantify EPC-specific gene expression, peripheral blood was incubated for 10 minutes with red blood cell lysing buffer (Sigma, Munich, Germany) and then centrifuged at 16,000 rpm for 20 seconds at 4°C. Total RNA isolation was performed using Trizol (Invitrogen) and cDNA was synthesized from each blood sample with the SuperScript II Reverse Transcriptase kit (Invitrogen) according to the manufacturer's instructions. Real-time PCR (25-μl reactions) using SYBR^® ^GreenER qPCR SuperMix Universal S (Invitrogen, USA) was performed in triplicate in the Mx3000p Real Time PCR System (Stratagene, USA). The following thermal cycling conditions were used: 10 sec at 95°C followed by 40 cycles of 15 sec at 95°C, 20 sec at 60°C, and 7 sec at 72°C. A no-template control (replacing RNA with water) was used as a negative control. Target gene expression was determined using the 2^-ΔΔCt ^method and normalized using β-actin as an internal control. To determine PCR amplification efficiency, standard curves were constructed using different concentrations of template cDNA for CD34, VEGFR-2, and β-actin. For all genes, the correlation coefficient of the standard curves was 0.98 or higher, and amplification efficiency was near 1.0.

The primer sequences used for real-time PCR were as follows: VEGFR2, 5'-CAC CAC TCA AAC GCT GAC ATG TA-3' and 5'-GCT CGT TGG CGC ACT CTT-3'; CD34, 5'-TTG ACA ACA ACG GTA CTG CTA C-3' and 5'-TGG TGA ACA CTG TGC TGA TTA C-3'; and β-actin, 5'-TCT GGC ACC ACA CCT TCT AC-3' and 5'-CTC CTT AAT GTC ACG CAC GAT TTC-3'.

### Plasma Assays

Blood levels of VEGF and MMP-9 were measured by enzyme-linked immunosorbent assay (ELISA) kit (R&D Systems, USA) according to the manufacturer's instructions.

### Statistical Analysis

Statistical analyses were performed with Statistical Package for Social Sciences 13.0 software (SPSS, USA). The Mann-Whitney U test and Student's *t*-test was used to compare variables between the two groups. Overall survival analyses were performed using the Kaplan-Meier method. Overall survival intervals were determined as the time period from initial diagnosis to the time of death. Because of skewed distributions, VEGF and MMP-9 levels are described using median values and ranges. EPC level and VEGF/MMP-9 levels were compared with the log-rank statistic. Data are expressed as mean ± standard error (SE). *P *< 0.05 was considered statistically significant.

## Results

### Numbers of EPCs in peripheral blood of ovarian cancer patients

**Figure 1:**
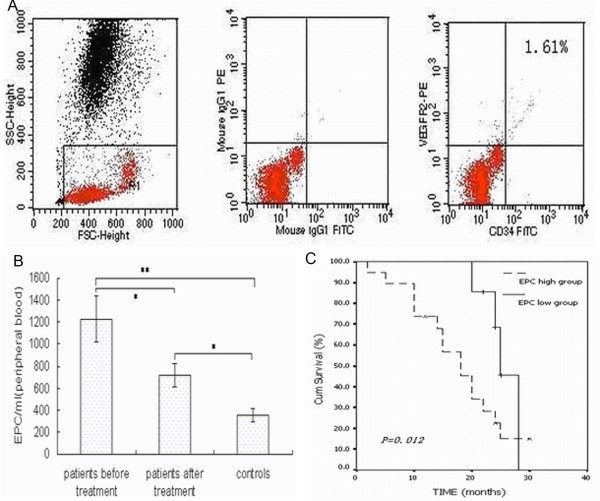
**(A) Representative flow cytometric analysis from a patient with ovarian cancer**. Left: flow cytometry gating. Middle: isotype negative control for flow-cytometry. Right: representative flow cytometric analysis for determining the number of CD34/VEGFR2 double-positive cells with a value of 1.61%. (B) Comparison of circulating EPC levels in ovarian cancer patients and healthy subjects. Data are expressed as mean ± SE  (**P < 0.01, *P < 0.05). (C) Kaplan-Meier overall survival curve of patients with ovarian cancer according to pre-treatment circulating EPCs numbers (*P *= 0.012). The cutoff value between low and high pre-treatment EPC levels was set at 945 EPCs/ml of peripheral blood (median value).

We determined the number of EPCs (CD34^+^/VEGFR2^+ ^cells) in the peripheral blood with flow cytometry. Figure 1A shows a representative flow cytometric analysis from a pre-treatment ovarian cancer patient (circulating CD34^+^/VEGFR2^+ ^cells, 1.61%). The percentage of double-positive cells (CD34^+^/VEGFR2^+^) was converted to cells per ml of peripheral blood using the complete blood count. The number of EPCs per ml in the peripheral blood of pre-treatment and post-treatment ovarian cancer patients (1260.5 ± 234.2/ml and 659 ± 132.6/ml) were higher than that of healthy controls (368 ± 34.5/ml; *P *< 0.01 and *P *< 0.05, respectively). Treatment significantly reduced the number of EPCs/ml of peripheral blood in patients (*P *< 0.05) (Fig. 1B).

After a median follow-up of 20.2 months, 26 of the 42 patients (62%) were alive and 16 patients (38%) had died from ovarian cancer. We established the pre-treatment EPC cutoff values (395, 670, 945, and 1220 per mL of peripheral blood; i.e., quartile numbers), which were tested for ability to predict disease outcome. Our results showed that low pre-treatment EPC levels (< 945/ml) were associated with longer survival compared with higher pre-treatment EPC levels (median survival time, 20.4 months, *P *= 0.012) (Fig. 1C).

### Relationship between circulating EPC levels and clinical behavior of ovarian cancer

Patient characteristics are summarized in Table 1. No difference in patient age or histologic subtype was observed between patient groups. The circulating EPCs levels in the peripheral blood of stage III and IV ovarian cancer patients (1450 ± 206.5/ml) was significantly higher than that of stage I and II patients (1023 ± 104.2/ml; *P *= 0.034). Furthermore, circulating EPCs levels in post-treatment ovarian cancer patients with larger residual tumors (≥ 2 cm) were significantly higher (875 ± 192.6/ml) compared with those of ovarian cancer patients with residual smaller tumors (523 ± 92.6/ml; *P *= 0.029).

**Table 1:** Clinical characteristics and circulating endothelial progenitor cells (EPC) levels of ovarian cancer patients

Clinical characteristic	Patients (n)	EPCs (per ml)	*P*
Age			NS
<43 years old	17	1154 ± 93.7	
≥43 years old	25	1205 ± 178.5	
Residual tumor size			0.029
<2 cm	22	523 ± 92.6	
≥2 cm	8	875 ± 192.6	
FIGO stage			0.034
I--II	8	1023 ± 104.2	
III--IV	34	1450 ± 206.5	
Histological subtype			NS
Serous	23	1165 ± 254.6	
Mucinous	13	1187 ± 223.7	
Endometrioid	6	1235 ± 198.4	
Therapy			NS
Chemotherapy	12	783.4 ± 162.5	
Surgery	30	605 ± 147.2	

FIGO, Federation of Obstetrics and Gynecology; NS, not significant. Data are expressed as mean ± SE.

We next sought to determine the relationship between treatment type and EPCs levels. Surgery and chemotherapy significantly reduced the number of EPCs per ml of peripheral blood. However, after treatment, EPCs levels in the 30 patients who underwent surgery (605 ± 147.2/ml) and EPCs levels in the 12 patients who received chemotherapy treatment (783.4 ± 162.5/ml) were still elevated compared with healthy controls (368 ± 34.5/ml; *P *= 0.046).

### EPC markers in peripheral blood of ovarian cancer patients determined by real-time RT-PCR

**Figure 2:**
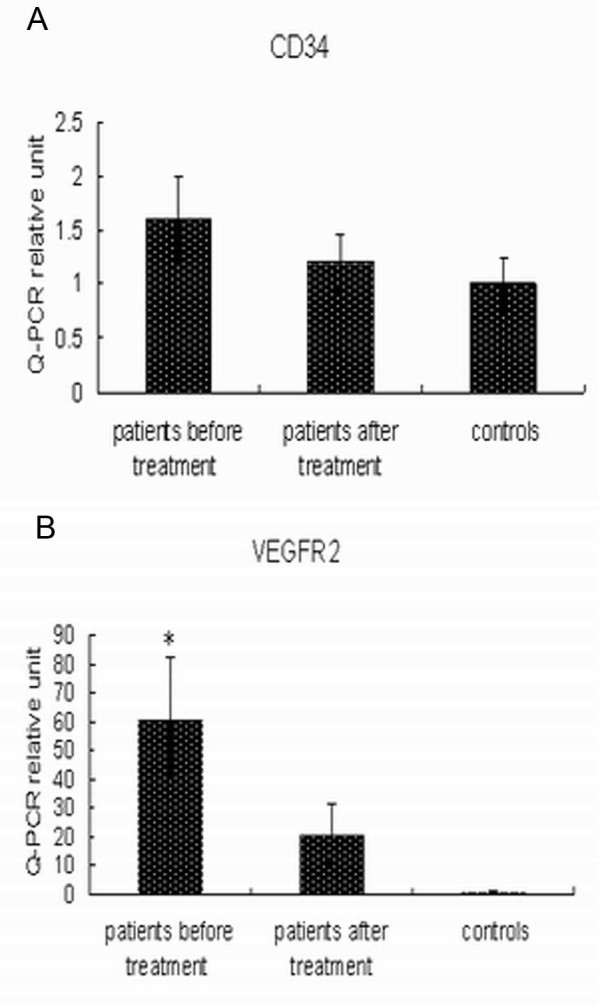
**Pre-treatment and post-treatment relative gene expression levels of (A) CD34 and (B) VEGFR2 were determined by real-time RT-PCR**. *P = 0.013, versus healthy subjects.

Peripheral blood CD34 and VEGFR2 mRNA levels were determined by real-time RT-PCR. Levels of CD34 were not significantly different in pre-treatment ovarian cancer patients compared with healthy controls (Fig. 2A), whereas VEGFR2 expression in pre-treatment ovarian cancer patients was 61-fold higher compared with healthy controls (*P *= 0.013) (Fig. 2B).

### Plasma levels of VEGF and MMP-9

**Figure 3:**
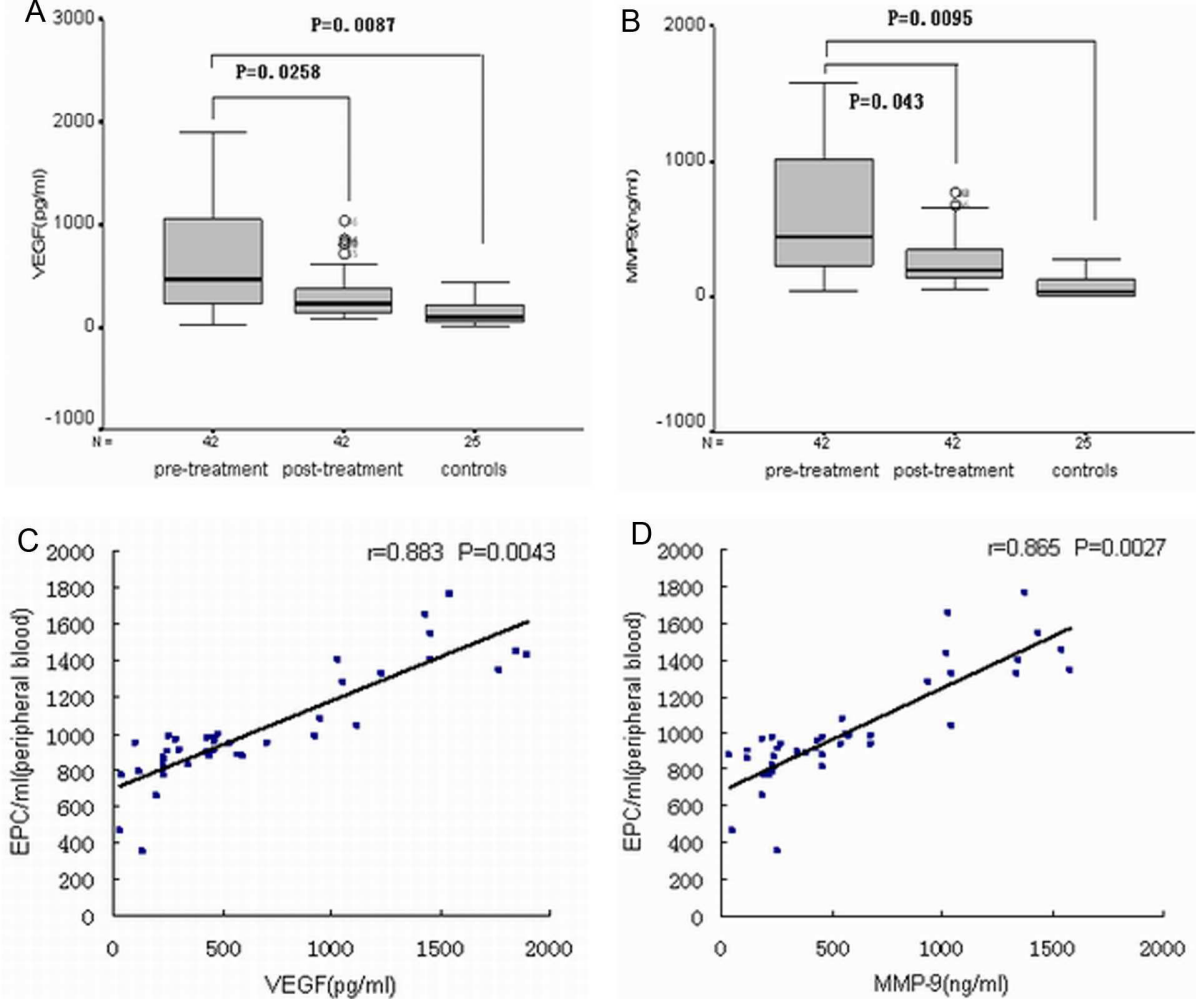
**Pre-treatment and post-treatment plasma levels of (A) VEGF (pg/ml) and (B) MMP-9 (ng/ml) in patients with ovarian cancer and healthy controls**. (C) Significant correlation was found between plasma VEGF and circulating EPC levels in patients with ovarian cancer (*P *= 0.0043, r = 0.883). (D) Significant correlation was found between plasma MMP-9 and circulating EPC levels for patients with ovarian cancer (*P *= 0.0027, r = 0.865).

We next compared plasma protein levels of VEGF and MMP-9 in pre-treatment and post-treatment ovarian cancer patients with those of healthy controls. For pre-treatment ovarian cancer patients, the median VEGF and MMP-9 protein concentrations were 609.1 pg/ml (range, 43.2-1845.2 pg/ml) and 404.3 ng/ml (range, 35.9-1623.6 ng/ml), respectively. VEGF and MMP-9 were present at detectable levels in healthy controls, but at lower concentrations, 64.4 pg/ml (range, 2.3-448.4 pg/ml) and 21.34 ng/ml (range, 0.8-335.6 pg/ml), respectively (*P *< 0.01). Treatment significantly reduced plasma protein levels of VEGF and MMP-9 to 180.5 pg/ml (range, 22.4-543.6 pg/ml) and 96.8 ng/ml (range, 12.8-415.9 pg/ml; *P *< 0.05) (Fig. 3A-B). Plasma concentrations of VEGF and MMP-9 and circulating EPC levels were correlated in pre-treatment ovarian cancer patients (*P *< 0.01, Fig. 3C-D).

## Discussion

EPCs are considered bone-marrow derived cells that migrate into the peripheral blood in response to cytokines such as VEGF [12]. In contrast to the ischemic condition, the role of circulating EPCs in tumor angiogenesis and growth is unclear. EPCs possess a high proliferation potential and have been found to be a potential marker for both neovascularization and response to antiangiogenic therapies [13]. The role of EPCs in cancer angiogenesis and growth deserves further investigation, especially in regard to their potential as markers to monitor disease progression or treatment response. However, to the best of our knowledge, the potential effect of circulating EPCs in the progression and angiogenesis of ovarian cancer has not been reported. In the present study, we investigated the potential utility of circulating EPCs as a marker for ovarian tumor progression, angiogenesis, and prognosis.

Previous studies demonstrated that EPCs levels in the peripheral blood of patients with breast cancer [14], non-small cell lung cancer [9], and lymphoma [15] were significantly higher compared with healthy volunteers. Similarly, we observed in the present study that the number of circulating EPCs was significantly higher in patients with ovarian cancer compared with healthy subjects. These findings support the results of animal studies regarding the mobilization and migration of bone marrow-derived EPCs via blood circulation into tumor neovasculature. Despite the small number of subjects in our study, we observed significant correlations between circulating EPCs levels and tumor stage and residual tumor size in ovarian cancer patients. This was consistent with a previous study that reported the relationship between increased EPC levels and more advanced stages of breast cancer [11].

We compared levels of EPCs in patients after surgery or chemotherapy treatment and found that both treatments reduced EPC levels, but not to the low level observed in healthy controls. Similarly, treatment was associated with a significant reduction in the levels of circulating EPCs in patients with lung cancer [9]. More importantly, follow-up revealed a significantly higher incidence of death from ovarian cancer in patients with high pre-treatment EPC levels compared with patients with low EPCs levels. These findings indicate a possible relationship between more aggressive ovarian cancer and higher circulating level of EPCs, suggesting that EPCs play a role in tumor growth and progression, thereby facilitating angiogenesis and metastasis.

We next attempted to characterize EPCs-specific markers CD34 and VEGFR2 in the peripheral blood of patients with ovarian cancer by real-time RT-PCR. Interestingly, pre-treatment VEGFR2 mRNA expression was increased in ovarian cancer patients compared with healthy controls, but no significant alteration was observed in CD34 mRNA levels. Elevated VEGFR2 levels may be due to variations in EPCs expression at different stages of cell development [12]; this surface receptor can be expressed on mature endothelial cells as well [16].

Accumulating evidence suggests that VEGF induces EPC mobilization from the bone marrow into circulation during tumor angiogenesis [17,18]. In the present study, soluble VEGF was significantly elevated in patients with ovarian cancer and was significantly reduced by treatment. Furthermore, circulating EPCs levels correlated with VEGF and MMP-9 plasma levels. However, the clinical relevance of these results is not completely understood. Recent studies reported that MMP-9 is important for stem and progenitor cell recruitment from the quiescent state into a permissive microenvironment following stress [19]. It is tempting to speculate that ovarian cancer tumor cells mobilize bone marrow-derived EPCs into circulation via VEGF and MMP-9 signaling; however, additional studies with larger patient groups are needed to elucidate these signaling pathways. Furthermore, circulating levels of VEGF and MMP-9 have been reported to be strongly associated with angiogenesis and ovarian cancer prognosis [20-22]. The present study provides additional evidence for the possible role of EPCs in ovarian cancer angiogenesis.

This study has some limitations. No unique marker for EPCs has yet been reported, and functional characterization of the rare putative EPCs population based on FACS phenotypes will be difficult to realize for a large dataset. Consensus on the exact nature of EPCs is needed to create a standardized, generally excepted methodology for enumeration of circulating EPCs [23,24]. Therefore, our descriptions of these cells may not be universally applicable, making comparisons with other published work difficult. Mature circulating endothelial cells (CECs) and hematopoietic progenitor cells may comprise part of the CD34^+^/VEGFR2^+ ^cells assessed in the present study. CECs are increased in the blood of cancer patients and correlate with tumor angiogenesis. Thus it is difficult to conclude that EPCs exclusively participate in ovarian cancer angiogenesis and growth. We speculate that EPCs induce endothelial sprouting through angiogenic growth factors, such as VEGF. With a better understanding of EPCs in the future, we can approach the role of EPCs in tumor progression and angiogenesis, and the effects of antiangiogenic agents in a more precise manner.

Our study demonstrates that EPCs levels are significantly increased in the blood of patients with ovarian cancer and are correlated with cancer stage and residual tumor size. Furthermore, treatment reduced circulating EPCs levels of patients. Although our data suggest a participation of EPCs in tumor growth and angiogenesis in ovarian cancer, it is not clear whether these cells are essential for this process. Further investigation is warranted for potential application of EPCs in monitoring disease progression and antiangiogenic drug efficacy, or as a target for ovarian cancer treatment.

## Conclusions

In conclusion, we believe that circulating EPCs may have potential as a biomarker for monitoring tumor progression and angiogenesis.

## Competing interests

The authors declare that they have no competing interests.

## Authors' contributions

YS participated in study design, carried out most of the experiments, and drafted the manuscript. LZ participated in collecting samples and manuscript preparation. QW conceived of the study, and participated in its design and coordination. WL assisted with cell culture. ZC participated in study design and statistical analysis. SX assisted with the critical revision of the manuscript. JB participated in study design and revised the manuscript. All authors read and approved the final manuscript.
